# Microelectromechanical System Resonant Devices: A Guide for Design, Modeling and Testing

**DOI:** 10.3390/mi15121461

**Published:** 2024-11-30

**Authors:** Carolina Viola, Davide Pavesi, Lichen Weng, Giorgio Gobat, Federico Maspero, Valentina Zega

**Affiliations:** 1 Civil and Environmental Engineering Department, Politecnico di Milano, Piazza Leonardo da Vinci 32, 20133 Milano, Italy; carolina.viola@polimi.it (C.V.); davide.pavesi@polimi.it (D.P.); lichen.weng@mail.polimi.it (L.W.); giorgio.gobat@polimi.it (G.G.); 2 Physics Department, Politecnico di Milano, Piazza Leonardo da Vinci 32, 20133 Milano, Italy; federico.maspero@polimi.it

**Keywords:** resonant MEMS, numerical modeling, design

## Abstract

Microelectromechanical systems (MEMSs) are attracting increasing interest from the scientific community for the large variety of possible applications and for the continuous request from the market to improve performances, while keeping small dimensions and reduced costs. To be able to simulate a priori and in real time the dynamic response of resonant devices is then crucial to guide the mechanical design and to support the MEMSs industry. In this work, we propose a simplified modeling procedure able to reproduce the nonlinear dynamics of MEMS resonant devices of arbitrary geometry. We validate it through the fabrication and testing of a cantilever beam resonator functioning in the nonlinear regime and we employ it to design a ring resonator working in the linear regime. Despite the uncertainties of a fabrication process available in the university facility, we demonstrate the predictability of the model and the effectiveness of the proposed design procedure. The satisfactory agreement between numerical predictions and experimental data proves indeed the proposed a priori design tool based on reduced-order numerical models and opens the way to its practical applications in the MEMS industry.

## 1. Introduction

Microelectromechanical systems (MEMSs) have been one of the most revolutionary technologies in recent years. The “Internet of Things” and, in general, the “Internet of Everything” are indeed paradigms of an increasingly connected world in which information is automatically transmitted by smart products like, e.g., phones, tablets, watches, glasses and cars. MEMS technology quickly led to an unprecedented miniaturization of sensors and actuators which are now playing an enabling role towards, e.g., artificial intelligence systems, robotics, autonomous mobility, remote patient monitoring and virtual reality, which indicates that their development in the next decades will be even faster with growing performance and applications.

Among different MEMS devices available so far in the literature and in the market, we will here focus on MEMS resonant devices. MEMS resonant devices are characterized by the presence of mechanical components that are kept at resonance through different actuation schemes. According to the employed actuation scheme, it is possible to distinguish between capacitive, piezoelectric and magnetic resonant devices. In this work, we will focus on capacitive MEMS resonant devices.

Capacitive MEMS resonant devices are widely employed for sensing, e.g., resonant accelerometers [1,2,3,4], gyroscopes [5,6] and as actuators, e.g., micromirrors [7,8] and microspeakers [9,10]. Recently, MEMS resonators [11] entered the market [12] of quartz oscillators as a possible solution to the increasing request of size reduction and integrability with the electronics and the other MEMS devices. Several examples of MEMS resonators fabricated either in single-crystal silicon [13] or polysilicon [14,15] are available in the literature.

To satisfy the ever-increasing request of better performance of MEMS resonant devices, a simple powerful and versatile design tool able to guide the electromechanical system optimization a priori, i.e., without resorting to parameters calibration on experimental data, is highly desired. Recent works addressed the numerical modeling of MEMS resonant devices [16,17,18,19], exhibiting complex and highly nonlinear dynamic responses due to, e.g., the interaction between membrane and bending regimes in slender beams or plates [20], to temperature variations [21], to internal contacts between surfaces at low distance [22] or to the electromechanical coupling induced by electrostatic actuation [23].

In this work, we propose a simple design tool able to predict, a priori and in real time, the nonlinear dynamic response of MEMS resonant devices of arbitrary geometry mainly making use of the commercial software COMSOL Multiphysics^®^ v.6.1. Thanks to its versatility and user-friendly nature, it represents a promising numerical tool for MEMS designers. To validate the proposed simulation, we fabricate and test a cantilever beam MEMS resonator exhibiting a nonlinear dynamic behavior. To further prove the potentiality of the numerical tool here developed, we employ it to design a MEMS ring resonator optimized to work in the linear regime.

MEMS fabrication has been constantly evolving in recent decades, progressing from early bulk micromachining and surface micromachining [24,25] to more advanced techniques using anisotropic etching such as deep reactive ion etching [26]. Advanced MEMS processes are now moving towards 3D architectures, utilizing multiple silicon layers to realize more complex MEMS designs [27,28,29]. Leading companies’ processes now include one polysilicon layer for routing electric signals and two silicon layers for constructing MEMS structures. Finally, a capping wafer is used to seal the device and control the pressure within the cavity.

Since the goal of this work is to provide a guideline for the design and modeling of MEMS resonant devices, we decided to use a relatively simple fabrication flow based on silicon-on-insulator substrates with a metal layer deposited on top. We shaped the metal layer and the silicon to obtain different MEMSs, as described in the relevant section. The advantage of this process is the small number of fabrication steps involved, making it feasible within a university facility yet transferable to more advanced MEMS processes.

This paper is organized as follows: in Section 2, the numerical model here proposed for the electromechanical design of MEMS devices is described in details. In Section 3, it is employed to reproduce the nonlinear dynamic response of a MEMS cantilever beam resonator, while in Section 4 it is used to design a ring resonator able to exhibit a linear dynamic response in the full admissible range of displacements. Conclusions and future perspectives are finally reported in Section 5.

## 2. Numerical Modeling

In this section, the guideline for a fast and accurate modeling of the damping sources present in MEMS resonant devices will be provided. Moreover, a numerical reduced-order technique able to efficiently predict the linear and nonlinear dynamics of MEMS resonant devices without resorting to long and computationally costly finite element simulations will be presented.

### 2.1. Quality Factor

Dissipation in a micromachined vibrating structure is measured through the so-called quality factor *Q* which is defined as follows:

(1) $$Q=\frac{2\pi W}{\Delta W}$$

where ΔW and *W* are the energy lost per cycle and the maximum value of energy stored in the device, respectively. Total dissipation can be determined as the sum of the different contributions, i.e., thermoelasticity, anchor losses and fluid damping, assuming perfect decoupling.

**Figure 1:**
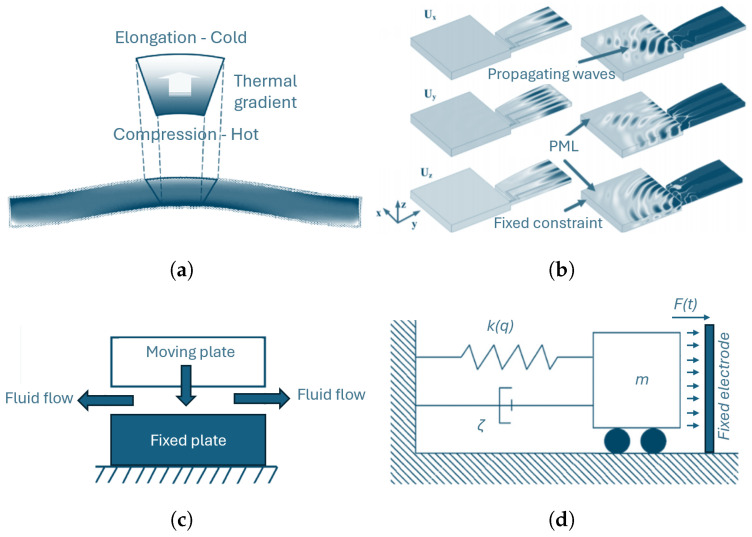
Schematic view of (**a**) thermoelastic damping, (**b**) anchor losses and (**c**) fluid damping. (**d**) One-degree-of-freedom schematization of the resonant device.

*Thermoelasticity* is an important solid dissipation mechanism for small-scale mechanical devices [30]. It is caused by the complex interaction of acoustic modes with thermally excited modes in the crystalline lattice. In Figure 1a, a schematic view of such interaction is reported for a clamped-clamped beam oscillating according to its first flexural mode. MEMS with significant bending deformations operating at low pressure are indeed typically dominated by thermoelastic losses. The analytical model proposed by Zener [31] makes it possible to estimate thermoelastic damping in simple beams undergoing flexural oscillations. The thermoelastic damping estimation for complex devices can be instead achieved by solving, according to a fully coupled approach [32] or following the staggered strategy [33], the fully coupled thermoelastic problem:

(2) $$\begin{array}{c} \rho \frac{\partial^{2}u}{\partial t^{2}}=\mathrm{div}\sigma ,\;\sigma =d\left(\epsilon -\alpha \left(T-T_{0}\right)I\right), \end{array}$$

(3) $$\begin{array}{c} \rho c_{h}\frac{\partial T}{\partial t}=\mathrm{div}\left(k\mathrm{grad}T\right)-\alpha T_{0}\frac{E}{1-2\nu }\frac{\partial \mathrm{tr}\epsilon }{\partial t} \end{array}$$

where u is the displacement field, σ is the stress tensor, d is the elastic tensor, ϵ is the strain tensor, ν is the Poisson’s ratio, $c_{h}$ is the specific heat, *k* is the thermal conductivity and *T* is the temperature field. In this work, the fully coupled thermoealastic problem in Equations (2) and (3) will be solved in COMSOL Multiphysics^®^ v.6.1. This choice, despite being less computationally efficient with respect to other methods based on the solution of the staggered thermoealastic problem [33], benefits from the user-friendly interface of a commercial software.

The second source of dissipation usually present in resonant MEMS devices is represented by *anchor losses*, which are due to the scattering of elastic waves into the substrate. Semi-analytical approaches for anchor losses dissipation estimation are available in the literature [30,34,35,36]. However, as observed for thermoelastic damping, anchor losses estimation through semi-analytical approaches is also only possible for very few simple geometries, while a complete and general numerical strategy is required if complex MEMS geometries are considered. Dissipative boundary conditions, and in particular the perfectly matched layer (PML) approach, are then attracting increasing attention in the literature [37,38]. The latter consists in a domain that is added to the model in proximity of anchors to mimic an open and non-reflecting infinite domain. From the implementation point of view, it corresponds to a coordinate transformation that continues the wave equation into complex coordinates, replacing propagating waves with exponentially decaying ones. In Figure 1b, the numerical settings employed for the simulation of anchor losses of a piezoelectric resonator are reported together with the predicted results.

Implementing a fully 3D PML analysis for the anchor losses estimation requires the solution of a large-scale generalized complex symmetric eigenvalue problem. If the mechanical structure vibrates in one of these modes, the quality factor is indeed given by the ratio between the real and imaginary parts of the eigenvalue, as detailed in [30] and experimentally validated in [38,39,40] for cantilever beams oscillating according to their flexural modes. In this work, anchor losses will be computed through COMSOL Multiphysics^®^ v.6.1, thus exploiting the user-friendly interface of a commercial code.

The last dissipation source usually present in MEMS resonant devices is represented by *fluid damping*, which is caused by the interaction of the moving mechanical components with the gases inside the package. In Figure 1c, a schematic view of the interaction between a parallel-plate capacitor and the surrounding fluid is reported for the sake of clarity. Depending on the pressure inside the package, it is possible to distinguish between different kinds of fluid damping. A large class of inertial sensors, e.g., accelerometers, works at pressures in the range of 0.5–1.0 bar. As a consequence, fluid inside the package can be treated as a continuum and standard Navier–Stokes models can be applied [41] to estimate fluid damping. If lower pressures *p* are instead exploited, the rarefied gas dynamics must be taken into account [42]. To identify the specific regime, the Knudsen number $K_{n}=\lambda /l$, where λ is the molecule mean free path and *l* is a typical dimension of the flux, must be evaluated. If $0.1<K_{n}<10$, the flow develops in the so-called transition regime and kinetic theories, e.g., the Boltzmann equation [30,43], must be solved to estimate fluid damping. If $K_{n}>10$, the flow enters the free-molecule flow regime and collisions between molecules can be neglected. A deterministic numerical model has recently been proposed in [44,45] and experimentally demonstrated in [46].

In this work, since resonant MEMS devices under evaluation work in near-vacuum condition, we focus on the free-molecular flow regime. To numerically simulate such damping source, we rely on the simplified, fast and operative simulation tool recently proposed for the prediction of gas damping occurring in MEMS devices working in near-vacuum conditions [47]. The tool, freely available online [47], is based on lookup tables pre-computed for the elementary blocks of typical resonant MEMSs, e.g., perforated masses, comb-finger, parallel plates, free-surfaces and sliding-surfaces, using the Boundary Integral Equation formulation. The fluid damping can be then estimated for every single elementary block and, under a perfect decoupling assumption, the total fluid damping can be numerically recovered. This represents a simplifying hypothesis that allows us to significantly reduce computational time without losing in terms of accuracy with respect to the monolithical approach presented in [44,45]. Note that such method is valid only under specific hypotheses on the natural frequency and on the geometry of the resonant devices, as detailed in [47].

Once the three main damping sources are numerically estimated as described above, the total quality factor can be computed as follows:

(4) $$Q_{TOT}={\left(\frac{1}{Q_{fluid}}+\frac{1}{Q_{TED}}+\frac{1}{Q_{AL}}\right)}^{-1}.$$

### 2.2. Dynamic Modeling

MEMS resonant devices dynamics can be modeled through appropriate reduced-order models by assuming the equivalence between the mechanical structure and a one-degree-of-freedom (1-dof) like the one schematized in Figure 1d. The most general nonlinear 1-dof model describing the dynamics of a MEMS resonant device reads as follows:

(5) $$m\ddot{q}\left(t\right)+\xi \dot{q}\left(t\right)+\left(k_{m,1}-k_{e,1}\right)q\left(t\right)+\left(k_{m,2}-k_{e,2}\right)q^{2}\left(t\right)+\left(k_{m,3}-k_{e,3}\right)q^{3}\left(t\right)=F\left(t\right)$$

where

- *m* is the modal mass of the system;
- ξ is the damping coefficient ($Q=\frac{m\omega }{\xi }$);
- *t* is the time variable;
- q(t), $\dot{q}\left(t\right)$, and $\ddot{q}\left(t\right)$ are the time-dependent amplitudes of the modal coordinate and of its time derivatives;
- $k_{m,1}$, $k_{m,2}$ and $k_{m,3}$ are the first-order, second-order and third-order mechanical stiffness coefficients;
- $k_{e,1}$, $k_{e,2}$ and $k_{e,3}$ are the first-order, second-order and third-order electrostatic stiffness components;
- F(t) is the amplitude of the time-dependent electrostatic force applied to the system.

The model in Equation (5) can be obtained assuming a modal projection of the displacement field u(t,x), with x spatial coordinate, including only the resonantly excited mode ϕ(x). This assumption gives the relationship between physical *u* and modal *q* coordinates, i.e., u(t,x)=q(t)ϕ(x). From now on, we assume a modal shape function normalized to unit, i.e., such that max(ϕ(x))=1. This assumption simplifies the relationship with the displacement field since its maximum value at a given time $t_{i}$ is given by $\bar{u}=max\left(u,t_{i}\right)=q\left(t_{i}\right)$. Once the eigenfunction ϕ(x) of the mode of interest is computed in COMSOL Multiphysics^®^ v.6.1 through a linear modal analysis, the modal mass *m* is computed as follows:

(6) $$m=\int_{\Omega }\rho \phi {\left(x\right)}^{2}dx,$$

where ρ is the material density and Ω is the volume of the MEMS resonant structure.

Electrostatic stiffness components and forcing term reported in Equation (5) can be estimated through integral equation-based models [23]. However, in case of simple actuation/detection schemes, e.g., parallel-plates or comb-fingers, and the hypothesis of moderate displacement of the mechanical structure, it is possible to apply an analytical formula as described in [30]. Despite such approach being less accurate than the one proposed in [23], it makes it possible to significantly reduce computational costs and be then employed in the real-time optimization process. Note that an accurate estimation of the electrostatic stiffness components through the method proposed in [23] can be performed a posteriori once the optimal geometry is identified.

In particular, the electrostatic force exerted between the deformed structure and a fixed parallel-plate capacitor can be approximated with a Taylor series expansion around the undeformed condition. These nonlinear contributions can be later projected into the modal subspace. Considering that the structures are modelled through an in-plane mode only, the projection can be written as a line integral along the mid-line of the moving structure facing the electrode. The resulting expression reads as follows:

(7) $$\begin{array}{cc} \; & \int_{0}^{L}\phi \left(x\right)\bar{F}\left(t\right)\;dx=\\ \; & \frac{\varepsilon_{0}V^{2}\left(t\right)}{2}\int_{0}^{L}\left(\frac{w\phi (x)}{g^{2}}+\frac{2w\phi {\left(x\right)}^{2}q\left(t\right)}{g^{3}}+\frac{3w\phi {\left(x\right)}^{3}q^{2}\left(t\right)}{g^{4}}+\frac{4w\phi {\left(x\right)}^{4}q^{3}\left(t\right)}{g^{5}}\right)\;dx \end{array}$$

where $\varepsilon_{0}$ is the dielectric constant in vacuum, *w* is the out-of-plane thickness of the silicon layer, V(t) is the time-dependent voltage difference between the two plates and *g* is the gap between parallel-plates at equilibrium.

Each term of Equation (7) can be computed analytically if the structure shows a simple geometry or numerically in case of complex mechanical structures.

Depending on the actuation/detection scheme employed in the experiments, it is possible to identify the nonlinear electrostatic stiffness components and the forcing term. In this work, as detailed in Appendix C, the moving mechanical structure is grounded, while a time-varying $V_{ac}cos\left(\omega t\right)$ signal is applied on the drive electrode, while a constant bias $V_{DC}$ is applied on the sense electrode, with ω external pulsation.

The forcing term F(t) can be expressed truncating the expansion to the zero-order term, as follows:

(8) $$F\left(t\right)=\frac{\varepsilon_{0}w}{2g^{2}}\left(\int_{0}^{L_{d}}\phi \left(x\right)\;dx\right)V_{ac}^{2}cos{\left(\omega t\right)}^{2}$$

where $L_{d}$ is the driving electrode length. Note that we only account for the $V_{ac}$-related contributions, thus neglecting the static force given by the $V_{DC}$ applied on the readout electrodes. This simplification is because the electrostatic expansion is performed around the undeformed configuration, using the eigenbases of the structure in the rest position. When a static load is applied, the induced stresses slightly change eigenmodes and eigenfrequencies. To avoid a dependence of the eigenspace with respect to the applied potential, i.e., full-electromechanically coupled problem, we here ignore this contribution. The effects of this assumption will be discussed in the Result Section.

On the other side, nonlinear electrostatic stiffness components depend only on $V_{DC}^{2}$ and read as follows:

(9) $$\begin{array}{c} k_{e,1}=\frac{\varepsilon_{0}w}{g^{3}}\left(\int_{0}^{L_{s}}\phi {\left(x\right)}^{2}\;dx\right)V_{DC}^{2} \end{array}$$

(10) $$\begin{array}{c} k_{e,2}=\frac{3\varepsilon_{0}w}{2g^{4}}\left(\int_{0}^{L_{s}}\phi {\left(x\right)}^{3}\;dx\right)V_{DC}^{2} \end{array}$$

(11) $$\begin{array}{c} k_{e,3}=\frac{2\varepsilon_{0}w}{g^{5}}\left(\int_{0}^{L_{s}}\phi {\left(x\right)}^{4}\;dx\right)V_{DC}^{2}, \end{array}$$

where $L_{s}$ is the readout electrode length.

Note that the proposed model for electrostatic actuation force and stiffness terms is valid for capacitive actuation/detection schemes. Different models must be considered in case of piezoelectric resonators which show a layered structure. Relevant examples can be found in [48,49].

Nonlinear mechanical stiffness components can be computed with different approaches. Among the nonlinear reduced-order models proposed in the literature, we mention the quadratic manifold built from modal derivatives [50,51], the nonlinear normal mode [52,53] and the direct parametrization method for invariant manifold (DPIM) [54,55] recently extended to electromechanical systems [56], the deep-learning based approaches [57,58] and the proper orthogonal decomposition [59].

Here, we will consider the implicit static condensation method. The implicit static condensation method [20,60], recently tailored for MEMS applications [17,23], relies on the evaluation of the nonlinear elastic force by statically forcing the structure with body forces proportional, through a coefficient β, to the eigenfunction ϕ(x). Such method is very accurate under the hypothesis of moderate transformations; moreover, it can be easily implemented in a commercial software, thus benefiting from its user-friendly interface.

A series of numerical static nonlinear analyses are run in COMSOL Multiphysics^®^ v.6.1 spanning the β space and computing the corresponding modal coordinate *q*, which in our case, coincides with the maximum displacement $\bar{u}$ of the MEMS resonant structure. The $\bar{u}\left(\beta \right)$ relation is numerically computed and then numerically inverted through fitting procedures, thus obtaining

(12) $$\beta \left(\bar{u}\right)=k_{m,1}\bar{u}+k_{m,2}{\bar{u}}^{2}+k_{m,3}{\bar{u}}^{3},$$

from which it is possible to identify the nonlinear mechanical stiffness components to be inserted in Equation (5) recalling that $\bar{u}=q$.

The described 1-DOF model can be used to predict the dynamics of resonant MEMS devices because their behavior is usually dominated by one activated eigenmode and all the other modes have well-separated frequencies. Moreover, nonlinear couplings between different modes are often avoided in operation.

Once nonlinear elastic and electrostatic stiffness components are determined, the amplitude of the resonant device oscillation $\bar{u}$ can be determined from Equation (5) through the multiple scale method [61]:

(13) $${\left(\frac{\bar{F}}{k_{1}}\right)}^{2}={\left(2\left(1-\frac{\omega }{\omega_{0}}\right)\bar{u}+\left(\frac{3k_{3}}{4k_{1}}-\frac{5}{6}{\left(\frac{k_{2}}{k_{1}}\right)}^{2}\right){\bar{u}}^{3}\right)}^{2}+{\left(\frac{\xi }{m\omega_{0}}\bar{u}\right)}^{2}$$

where $\omega_{0}$ is the natural frequency of the device defined as $\sqrt{k_{1}/m}$, $k_{1}=k_{m,1}-k_{e,1}$, $k_{2}=k_{m,2}-k_{e,2}$, $k_{3}=k_{m,3}-k_{e,3}$ and ω is the frequency of the electrostatic force having amplitude $\bar{F}$. An alternative numerical approach able to compute the nonlinear solution of Equation (5) is, for example, the continuation technique available in the MATLAB R2022a MATCONT package [62].

The goal of this work being to compare numerical results achieved in this section with experimental data, frequency response curves of the two resonators will be shown in terms of capacitance variation read on the readout electrode. To move from the frequency response here computed in terms of maximum displacement to the one shown in Section 3.3 and Section 4.3, the following relation is employed:

(14) $$\Delta C\left(t\right)=\frac{\varepsilon_{0}w}{g^{2}}\int_{0}^{L_{s}}\left(\phi \left(x\right)q\left(t\right)+\frac{\phi {\left(x\right)}^{2}q^{2}\left(t\right)}{g}+\frac{\phi {\left(x\right)}^{3}q^{3}\left(t\right)}{g^{2}}\right)\;dx$$

Note that temperature is considered constant and equal to $T=25^{\circ }$ in this work. If temperature effects want to be considered in the modeling, it is necessary to follow the procedure proposed in [21,33] to update materials properties and consequently obtain a different dynamic response for every temperature value in the range of interest.

## 3. Cantilever Beam Resonator

In the present section, a MEMS cantilever beam resonator showing a nonlinear dynamic behavior is employed to validate the described numerical model.

### 3.1. Mechanical Design

**Figure 2:**
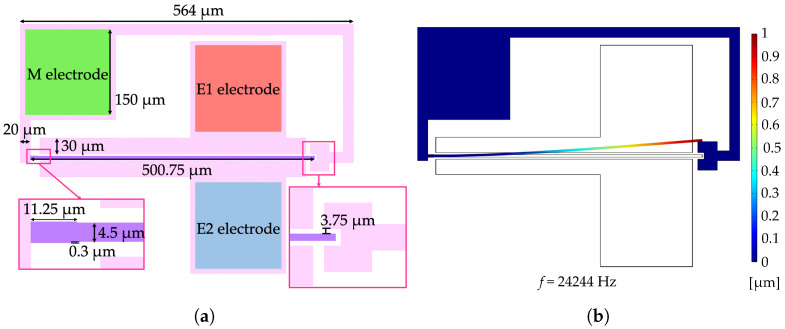
(**a**) Cantilever beam resonator geometry with dimensions. Parallel-plates electrodes employed for the capacitive actuation/readout are labeled M, E1 and E2. (**b**) Eigenfrequency and modal shape of the cantilever beam resonator computed in COMSOL Multiphysics^®^ v.6.1. The normalized displacement field is shown in colors.

In Figure 2a, the general scheme of the resonator is shown where the elongated beam of in-plane dimension 500.75 μm × 4.5 μm is clamped to the substrate on one side and free to oscillate on the other side.

Two fixed electrodes are located at the two sides of the resonator at a nominal gap of 3.75 μm to guarantee electrostatic actuation and capacitive readout. Three electric contacts are also provided, namely M, E1 and E2, to apply different voltages to the moving structure and the two fixed electrodes, respectively, during the experimental campaign.

To improve robustness of the device during fabrication, a stopper (see close-up view in Figure 2a), short-circuited with the beam resonator, is placed on the free-end of the resonator at a distance of 3.75 μm and a reinforcement (see close-up view in Figure 2a) is added in the most exposed part of the resonator, i.e., close to the clamp.

The cantilever beam resonator is sized to resonate at 24,244 Hz according to its first flexural mode reported in Figure 2b. Numerical simulations were run in COMSOL Multiphysics^®^ v.6.1. Note that a nominal over-etch of 1 μm is taken into account.

### 3.2. Numerical Modeling

The three sources of damping are simulated employing the methods explained in Section 2.1. The results are summarized in Appendix A.

The beam resonator, tested at low pressure but not in vacuum condition as clearly described in Section 3.3, shows a quality factor dominated by fluid damping $Q_{fluid}$ as summarized in Table 1.

In Table A1, the coefficients present in Equation (5) are summarized.

As expected from the literature [16], geometric nonlinearities in the beam resonator result in an hardening behavior ($k_{m,3}>0$). Electrostatic nonlinearities are instead softening in nature. Depending on the actuation/detection voltage, the overall dynamic response of the beam resonator will show a hardening or a softening behavior. Note that the beam resonator behavior is dominated by the lowest frequency in-plane eigenmode and all the other modes have well-separated frequencies; moreover, we do not observe any nonlinear coupling in the experiments. The one-degree-of-freedom approximation of Equation (5) is then enough to reproduce the dynamic behavior of the MEMS cantilever beam resonator.

### 3.3. Experimental Tests

The MEMS cantilever beam is fabricated as described in Appendix B and tested according to the set-up described in Appendix C.

**Figure 3:**
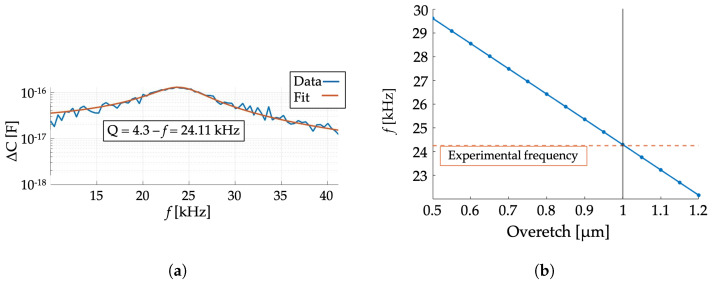
(**a**) Experimental frequency response curve of the cantilever beam resonator measured in air. Identified eigenfrequency and quality factor are reported in the inset. (**b**) Eigenfrequency numerical estimation as a function of the over-etch in comparison with the experimental value (orange dotted line).

The first experimental campaign performed in air is employed to identify the average over-etch suffered by the fabricated structure. A $V_{DC}$ of 5 V is applied on electrode E1, while a $V_{ac}$ of 5 V is applied on electrode E2. In Figure 3a, the frequency response curve obtained with this setup is reported: an experimental eigenfrequency of 24.11 kHz and a quality factor of 4.3 are identified through a proper fitting (orange curve). The resonance frequency numerically computed as a function of the over-etch (blue line) is reported in Figure 3b, together with the experimental results (orange horizontal line): the identified over-etch is 1 μm, which is in agreement with the one assumed in the design phase.

To validate the 1-dof nonlinear model described in Section 2.2, experimental frequency responses are obtained in near-vacuum conditions by employing a custom-build vacuum chamber. Note that the nominal base pressure is lower than $10^{-3}$ mbar, but it can take some time to reach the target value, thus leading to slightly different fluid damping even if the same actuation condition is considered.

**Figure 4:**
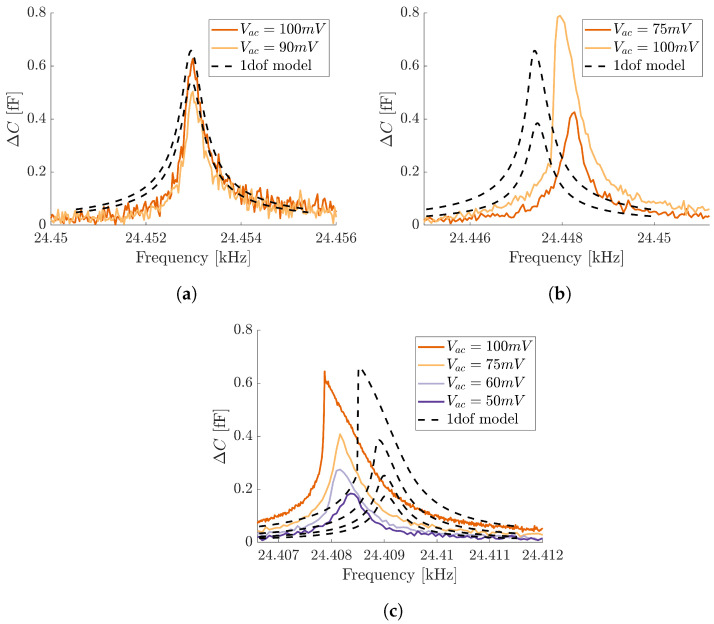
Comparison between experimental frequency response curves (continuous line) and numerical predictions (dashed line) for different actuation voltages $V_{ac}$. The readout DC voltage is instead kept fixed and equal to (**a**) $V_{DC}=0.5$ V, (**b**) $V_{DC}=1$ V and (**c**) $V_{DC}=2.5$ V.

In Figure 4, experimental curves are compared with numerical predictions for different actuation/readout voltages reported in the legend of the graph.

The cantilever beam resonator, when excited at high voltages, enters the nonlinear regime showing a softening response, i.e., the frequency response curve bends to the left. Such behavior is due to the presence of relevant electrostatic stiffness contributions, which compensate for the mechanical hardening effect until overcoming it.

The proposed numerical model correctly catches the overall softening behavior, and small discrepancies in the order of $2.45\times 10^{-3}$% in terms of natural frequency and 20% in terms of maximum amplitude, in the worst case scenario, evident from Figure 4, derive from the approximations conducted in the model. In particular, we employ a uniform over-etch for the full mechanical structure. This is a strong assumption considering fabrication imperfections that can arise in such process. Moreover, in the model, we do not consider any pre-deflection of the cantilever beam resonator induced by fabrication pre-stresses and/or by the DC voltage applied on the readout electrode. Such effects, which are reasonably present in the fabricated device, can be included in the model in order to achieve a better agreement, as achieved in [48].

**Figure 5:**
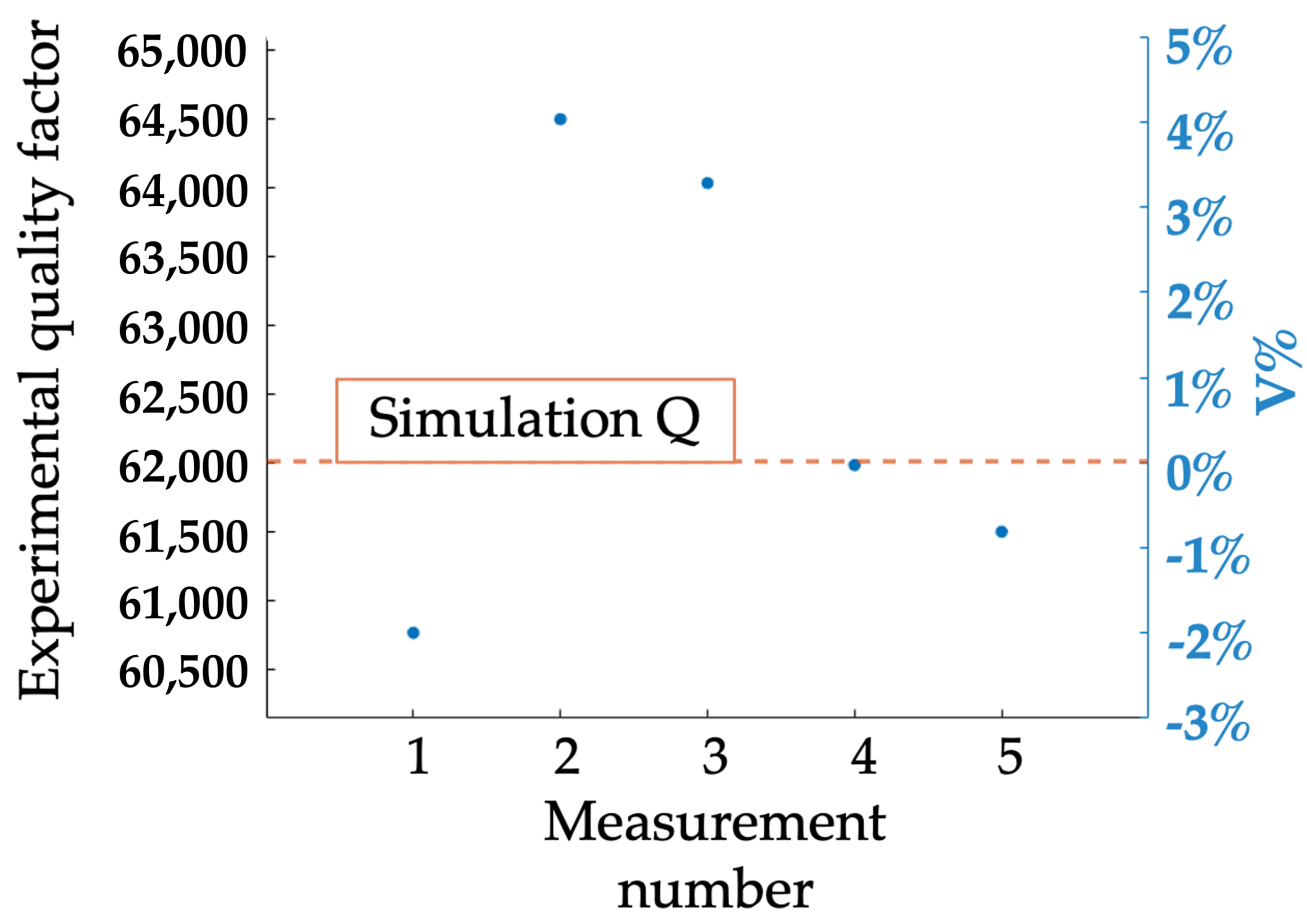
Experimental quality factors versus numerical prediction. The *Q* percentage variation is computed as $V_{\%}=\frac{Q_{exp}-Q_{sim}}{Q_{sim}}$, where $Q_{exp}$ is the experimental value, and $Q_{sim}$ is the simulation value.

To further validate the simulation, we perform a comparison between the experimental quality factor obtained in low-pressure conditions and the simulation reported in Section 3.2. In Figure 5, the quality factors identified through the half-power bandwidth technique on the experimental linear dynamic responses measured for $V_{DC}$ = 0.4 V, 0.5 V, 1 V, 2.5 V, 2.5 V and $V_{ac}$ = 0.007 V, 0.9 V, 0.05 V, 0.06 V, 0.1 V, respectively, are reported in dots together with the numerical prediction dotted orange line. A satisfactory agreement between simulation and experiment is achieved: the identified error is indeed within the 5% which is fully compatible with the uncertainties on the pressure value inside the chamber, material thermal parameters and over-etch distribution that strongly influence fluid damping, thermoelastic dissipation and anchor losses, respectively.

## 4. Ring Resonator

Aware of the very promising results achieved on the cantilever beam resonator, we here employ the modeling strategy proposed in Section 2.2, to design a ring resonator with a linear frequency response in the full admissible displacement range, i.e., around one-third of the gap.

### 4.1. Mechanical Design

**Figure 6:**
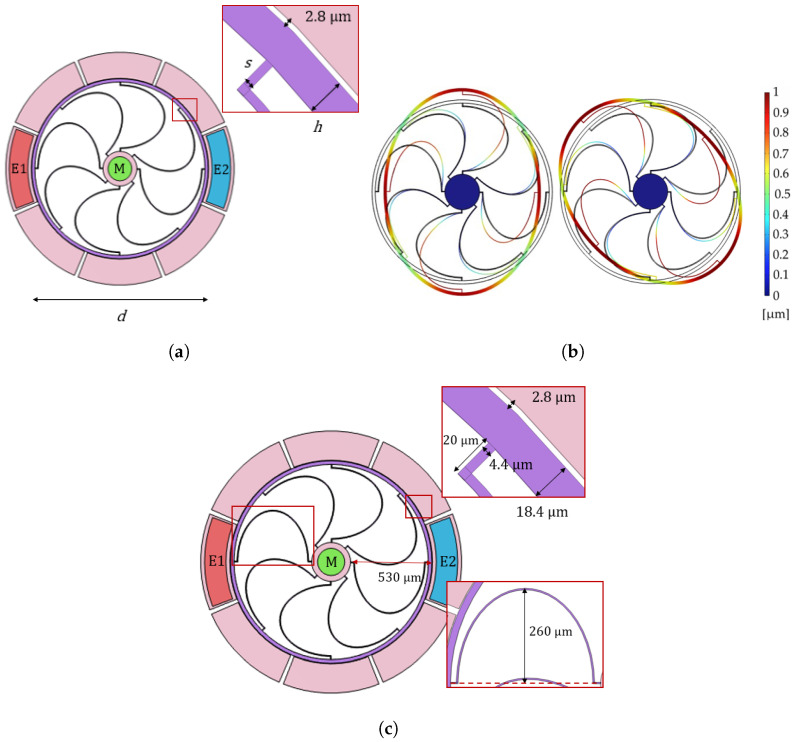
(**a**) Ring geometry with dimensions. Parallel-plates electrodes employed for the capacitive actuation/readout are labeled M, E1 and E2. Eigenfrequency and modal shapefunction of the (**b**) 0°−mode and the 45°−mode of the ring resonator computed in COMSOL Multiphysics^®^ v.6.1. The normalized displacement field is shown in colors. (**c**) Geometric dimensions of the optimized ring resonator.

The mechanical structure of the ring resonator is schematically shown in Figure 6a, where the main geometric variables are also reported for the sake of clarity.

The idea is to run a parametric optimization by considering the nonlinearity coefficient $NL_{\%}$ as qn objective function to minimize. The nonlinear coefficient is here defined as follows:

(15) $$NL_{\%}=\frac{max\left(u_{L}\right)-max\left(u_{NL}\right)}{max(u_{L})}\times 100$$

where $u_{L}$ and $u_{NL}$ are the ring resonator displacements computed for different actuation voltages by neglecting and considering nonlinearities, respectively. The admissible range of actuation voltages is defined such as pull-in is not reached. The gap between the ring resonator and the electrodes being defined by the process, we here consider $V_{ac}$ amplitudes which correspond to displacement amplitudes equal to one-third of the gap.

Geometric parameters, shown in Figure 6a, are spanned in the admissible range dictated by the fabrication process, described in Appendix B, and the $u_{L}$ and $u_{NL}$ relative to the two-theta modes shown in Figure 6b are computed for every set of parameters. The 1-DOF model of Equation (5) is used to estimate the nonlinear displacement field $u_{NL}$ of each mode, separately. Despite the fact that the ring resonator dynamic behavior may lead to some interactions with the (almost) degenerated in-plane modes, in the present implementation, the frequencies of the modes are too far apart to lead to nonlinear couplings. The geometry is indeed kept symmetric even if the single-crystal silicon is not isotropic. Furthermore, as confirmed in the experiment section, we do not observe any saturation phenomena, i.e., one of the effects of degenerated mode coupling [57,58,63]; thus, a 1-DOF modeling is sufficient for the scope of this paper. Independently on the values assumed by the geometric parameters, both geometric and electrostatic nonlinearities are softening in nature. An overall softening dynamic response is then expected when the nonlinear regime is entered. From the optimization procedure, it has been observed that, increasing the spring thickness (*s* in Figure 6a), the nonlinear coefficient slightly increases. However, the biggest influence on the nonlinear coefficient is provided by the ring thickness (*h* in Figure 6a): bigger *h* results in higher nonlinearities.

Aware of the outcome of the parametric optimization process, structural parameters of the optimized ring resonator are finally identified such as to have spurious modes as distant as possible from the two in-plane elliptical modes, while still preserving a good linear behavior. The ring thickness *h* indeed also determines the relative position of the two-theta modes of interest with respect to the lower in-plane spurious modes and higher out-of-plane modes. In particular, increasing *h*, the frequency difference with lower and higher spurious modes increases and decreases, respectively. Moreover, increasing the spring thickness *s*, 0°−mode and 45°−mode become closer in frequency to lower in-plane spurious modes. Spring thickness is therefore set equal to the minimum value allowed by the process. The influence of the number of the rounded springs is also investigated since it also governs the position in frequency of the different modes.

The optimized ring resonator shown in Figure 6c consists of a circular ring of diameter 1060 μm linked to a central anchor through eight semi-elliptical springs of in-plane thickness 4.4 μm. Eight equally-sized electrodes are positioned outside the circular ring at a distance of 2.8 μm to allow for the electrostatic actuation and capacitive readout. Note that a nominal over-etch of 1.4 μm is here already taken into account. Such value is identified from a visual inspection of the SEM of the outer ring of the fabricated ring resonator (Appendix B). However, different over-etches are expected in different parts of the structure due to the fabrication process.

The optimized ring resonator oscillates according to the two in-plane elliptical modes shown in Figure 6b, namely 0°−mode and 45°−mode, at frequencies 70,358 Hz and 74,574 Hz, respectively. The closest spurious modes consist in an out-of-plane movement of the springs (at 59,700 Hz) and of the external ring (at 84,100 Hz). Degenerate in-plane spurious modes are also present: they consist in pure in-plane translations of the ring and resonate at 14,700 Hz. Resonant frequencies are here estimated through modal analysis run in COMSOL Multiphysics^®^ v.6.1.

As for the cantilever beam resonator, also in this case, three electric contacts are provided, namely M, E1 and E2, to guarantee different testing conditions.

### 4.2. Numerical Modeling

Quality factors of the two-theta modes of the optimized ring resonator are estimated employing the methods explained in Section 2.1. In particular, $Q_{TED}$s of 20,247 and 17,284 are obtained for the 0°−mode and 45°−mode, respectively. The different thermoelastic quality factors estimated for the two nominally identical modes of the ring resonator are due to the orthotropic nature of the single-crystal silicon employed for fabrication (see Appendix B). Anchor losses ($Q_{AL}$) were also estimated through the PML technique in COMSOL Multiphysics^®^ v.6.1, but since the results are orders of magnitudes higher than $Q_{TED}$, they are here neglected for the sake of simplicity. Fluid damping is also neglected, the ring resonator being tested in high-vacuum conditions.

Coefficients of the one-degree-of-freedom model obtained as explained in Section 2.2 are summarized in Appendix A.

**Figure 7:**
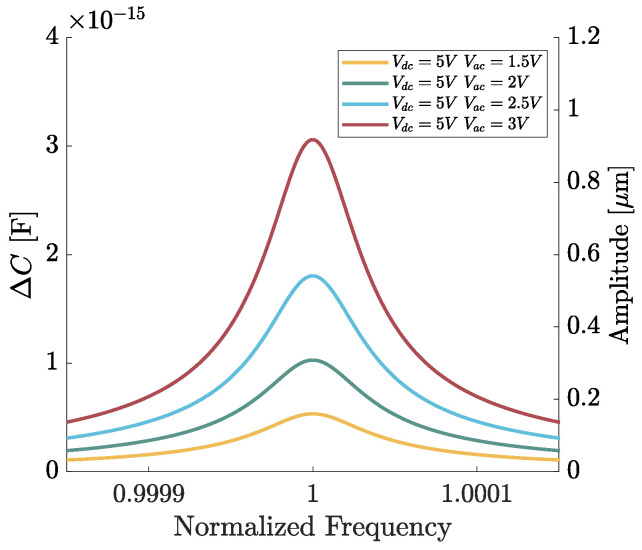
Frequency responses of the optimized ring resonator numerically simulated for $V_{ac}$ spanning from 1.5 V to 3 V. $V_{DC}$ is kept equal to 5 *V*.

In Figure 7, the frequency responses of the optimized ring resonator numerically simulated for $V_{ac}$ spanning in the admissible range (up to 3 V) are reported. They indeed correspond to a maximum displacement of 0.9 μm, as shown in the right axis of Figure 7. The softening behavior is below $10^{-5}$% for maximum admissible displacements equal to 0.9 μm, thus demonstrating the effectiveness of the proposed a priori design optimization.

### 4.3. Experimental Tests

The ring resonator is characterized in a vacuum probe station (ARS PS-L Flow Cryostat, Advanced Research Systems 7476 Industrial Park Way, Macungie, PA 18062 (USA)) at a pressure below $10^{-4}$ mbar. A $V_{DC}$ of 5 V is applied on electrode E1, while $V_{ac}$ of 1.5 V is applied to the E2 electrode.

Three nominally identical ring resonators are characterized in the linear regime, i.e., R1, R2, R3. Results in terms of natural frequency and quality factor are summarized in Table 2. Experimentally measured frequencies are comparable to the ones obtained from simulations: the small differences are related to over-etch variations with respect to the nominal one considered during the design.

**Figure 8:**
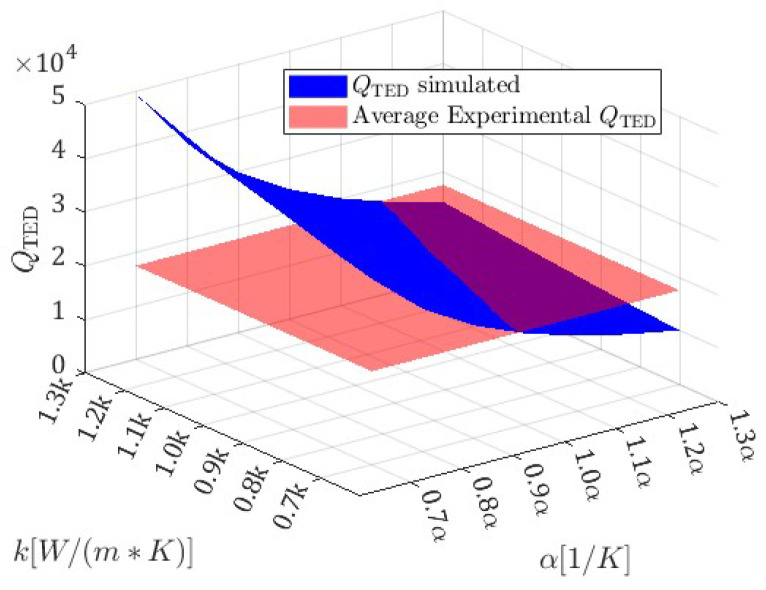
$Q_{TED}$ simulated in COMSOL Multiphysics^®^ v.6.1 by sweeping linear coefficient of thermal expansion α and thermal conductivity *k* (blue surface). The average value of experimental $Q_{TED}$ is also reported for comparison (red plane).

The experimental quality factor, obtained through fitting of experimental results, is slightly lower than the predicted one. This can be justified by the fact that $Q_{TED}$, which is the main dissipation source for the ring resonator under study, strongly depends on material thermal properties. In the model here proposed, linear expansion coefficient $\alpha =2.53\times 10^{-6}$1/K, heat capacity at constant pressure $C_{p}=702.93$ 1/(Kg K) and thermal conductivity k=148 W/(m K) for single-crystal silicon are obtained from [33] and can differ from the actual ones. In Figure 8, quality factors $Q_{TED}$ computed through COMSOL Multiphysics^®^ v.6.1 for the 0°−mode by sweeping values of α and *k* in a reasonable range (blue plane) are reported for the sake of clarity in comparison with the experimental one (red plane). It is then evident that a small variation in such material parameters can justify the discrepancy between experiments and numerical predictions. The effect of $C_{p}$ variation on $Q_{TED}$ is negligible. The same considerations are also valid for $Q_{TED}$ discrepancies in 45°−mode.

**Figure 9:**
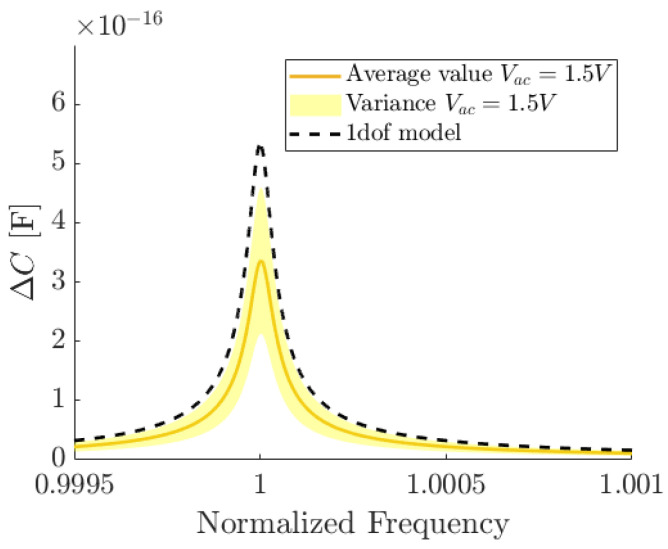
Comparison between the average value of experimental frequency response curves (continuous line) and numerical prediction (dashed line) for 45°−mode. R1, R2 and R3 are tested at $V_{DC}=5$ V and $V_{ac}=1.5$ V: shaded area represents the variance in experimental data.

A comparison between experimental and numerical frequency responses is finally shown in Figure 9 and a satisfactory agreement is found. Differences both in terms of resonance frequencies and in capacitance variations can be related to over-etch non-uniformities and to the assumptions made in the one-degree-of-freedom model.

## 5. Conclusions

A fast and efficient simulation tool able to predict a priori and in real time the dynamic response of MEMS capacitive devices has been proposed and validated on an MEMS cantilever beam resonator. A ring resonator able to operate in the linear regime is then designed through the proposed simulation tool. The two MEMS resonators were also fabricated and experimentally tested. A satisfactory agreement between numerical predictions and experimental data is found, thus proving the effectiveness of the proposed simulation tool.

The numerical tool here proposed represents a great step further towards the automatization of the MEMS design process. It indeed balances the versatility, user-friendly nature, predictability and real-time computation ability with acceptable engineering assumptions. The main challenge in the creation of such a design tool is indeed to be able to keep computational costs low while preserving the accuracy and the a priori characteristic of more complex and less user-friendly approaches.

Future work will be addressed to improve the agreement between experiments and numerical predictions while keeping computational costs low. As an example, a non-uniform over-etch and pre-stresses induced by both the fabrication process and the DC voltage will be accounted for in the model. To do so, an extensive experimental campaign will also be carried out to improve and characterize the fabrication process.

## Figures and Tables

**Table 1:** Numerically estimated quality factors of the beam resonator. $Q_{TED}$ and $Q_{AL}$ were obtained via COMSOL Multiphysics^®^ v.6.1, while $Q_{Fluid}$ was obtained using the tool proposed in [47].

	Beam Resonator
$Q_{TED}$	$1.82\times 10^{6}$
$Q_{AL}$	$3.24\times 10^{9}$
$Q_{fluid}$	64,197
$Q_{TOT}$	62,013

**Table 2:** Resonance frequency $f_{0}$ and quality factor *Q* obtained from experimental data.

	R1	R2	R3
$f_{0}$ (0°−mode)	70,745	−	70,745
*Q* (0°−mode)	17,300	−	17,450
$f_{0}$ (45°−mode)	74,991	75,010	74,854
*Q* (45°−mode)	15,398	14,878	15,531

**Figure A1:**
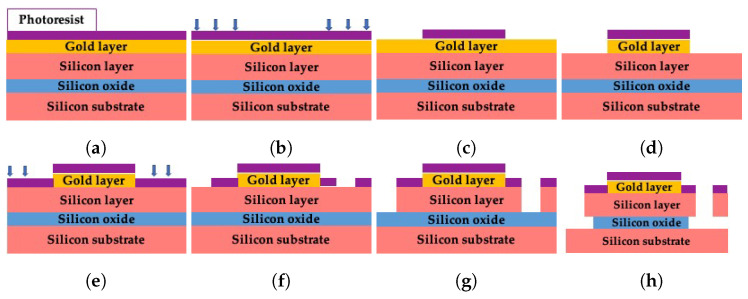
(**a**) Spin coating and soft baking of the photoresist; (**b**) Maskless photolithography for the gold layer; (**c**) Photoresist development; (**d**) Gold layer etching; (**e**) Spin coating and soft baking of the photoresist, and silicon layer maskless photolithography; (**f**) Photoresist development; (**g**) DRIE for silicon layer; (**h**) Release process via HF.

**Figure A2:**
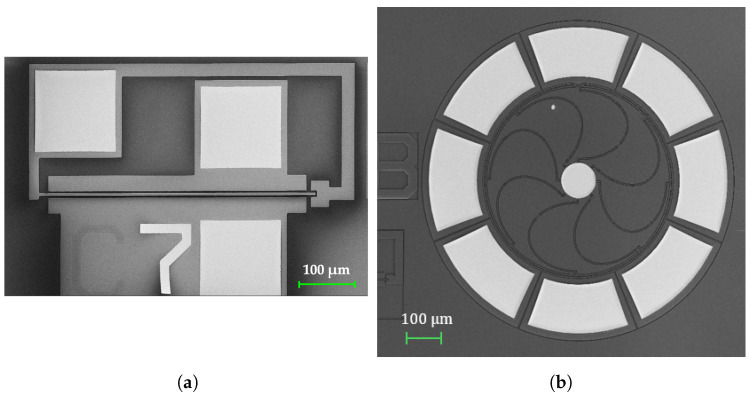
SEM image of the (**a**) cantilever beam and (**b**) ring resonators.

**Figure A3:**
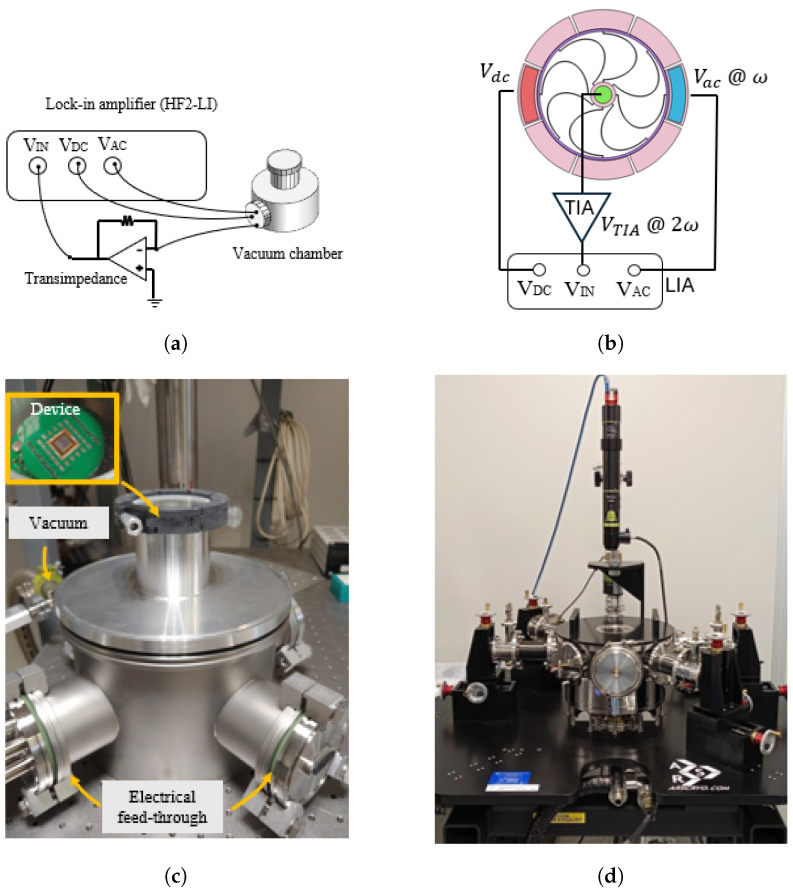
Experimental set-up employed for the electromechanical characterization of the cantilever beam and the ring resonator. (**a**) Schematic view of the set-up. Lock-in and transimpedence amplifier are connected to the vacuum chamber where the MEMS is placed. (**b**) Schematic view of the connections. AC and DC voltage are applied to the electrodes of the MEMS structure, while the TIA virtual ground is connected to the mass M. (**c**) Image of the vacuum chamber used for testing the cantilevers. Devices are bonded on a PCB carrier as shown in the inset. (**d**) Image of the vacuum probe-station used for testing gyroscopes.

## Data Availability

The original contributions presented in the study are included in the article, further inquiries can be directed to the corresponding author.

## Appendix A. One-Degree-of-Freedom Model Coefficients

**Table A1:** Mechanical nonlinear stiffness components computed through the implicit static condensation technique. Electrostatic nonlinear stiffness terms as function of the applied voltages.

	Beam	Ring 0°−Mode	Ring 45°−Mode
*m* [ng]	13.068	375.26	366.57
$k_{m,1}$ [N/m]	0.3242	73.38	81.38
$k_{m,2}$ [N/m^2^]	$-3\times 10^{-12}$	$1.69\times 10^{4}$	$-3.67\times 10^{3}$
$k_{m,3}$ [N/m^3^]	$1\times 10^{7}$	$-7.33\times 10^{10}$	$-9.66\times 10^{10}$
$k_{e,1}$ [μN/μm]	$7.56\times 10^{-4}\;V_{DC}^{2}$	$6.51\times 10^{-4}\;V_{DC}^{2}$	$9.33\times 10^{-4}\;V_{DC}^{2}$
$k_{e,2}$ [μN/μm^2^]	$7.48\times 10^{-5}\;V_{DC}^{2}$	$3.09\times 10^{-4}\;V_{DC}^{2}$	$5.28\times 10^{-4}\;V_{DC}^{2}$
$k_{e,3}$ [μN/μm^3^]	$8.34\times 10^{-6}\;V_{DC}^{2}$	$1.24\times 10^{-4}\;V_{DC}^{2}$	$2.69\times 10^{-4}\;V_{DC}^{2}$
*F* [μN]	$3.04\times 10^{-4}\;V_{ac}^{2}$	$5.10\times 10^{-4}\;V_{ac}^{2}$	$6.11\times 10^{-4}\;V_{ac}^{2}$

## Appendix B. Fabrication

The fabrication of the devices is performed at PoliFAB, the cleanroom of Politecnico di Milano, starting from an existing substrate. The starting substrate is a silicon on insulator having 10 μm or 5 μm of n-type doped silicon, for the beam-resonator and the ring-resonator, respectively, on top of 1 μm of silicon oxide. The silicon is grown epitaxially starting from a seed layer of mono-crystal silicon having (100) orientation. The 〈100〉-single-crystal silicon material parameters presented in [64] were employed here. Density ρ is assumed equal to 2330 Kg/m^3^, while the stiffness matrix *C* reads as follows:

(A1) $$C=\left[\begin{array}{cccccc} c_{11} & c_{12} & c_{12} & 0 & 0 & 0\\ c_{12} & c_{11} & c_{12} & 0 & 0 & 0\\ c_{12} & c_{12} & c_{11} & 0 & 0 & 0\\ 0 & 0 & 0 & c_{44} & 0 & 0\\ 0 & 0 & 0 & 0 & c_{44} & 0\\ 0 & 0 & 0 & 0 & 0 & c_{44} \end{array}\right]$$

where $c_{11}$ = 165.7 GPa, $c_{12}$ = 63.9 GPa and $c_{44}$ = 79.6 GPa.

On top of the silicon, a layer of gold is deposited together with a barrier layer to avoid inter-diffusion of the gold into silicon and to promote adhesion. The main process steps to obtain the MEMS from this substrate are reported in Figure A1. Steps (a) to (d) show the patterning of the metal layer. A photoresist mask is deposited by means of spincoating before exposing the desired pattern using a maskless aligner (model MLA100 from Heidelber Instruments Mikrotechnik GmbH, Mittelgewannweg 27, 69123 Heidelberg, Germany).

The sample is then dipped into a developer to remove the photoresist mask that was exposed during lithography. The photoresist mask is used to protect and define the contacts during the metal etching. Chemical etching is used to remove the gold layer and its adhesion layer from the silicon substrate (d). After patterning the metal contacts, a second litographic step is performed in order to define the MEMS structures. Following litography, Si-etching is performed with Dry Reactive Ion Etching with the Plasmalab 100 tool from Oxford Instruments Plasma Technology, 56.8 mi, Severn Beach, Bristol, UK Govier Way, Western Approach Distribution Park, Severn Beach, Bristol BS35 4GG, adopting a BOSCH-like process. Finally, dry release process (h) is used to remove the wafer’s sacrificial layer. This step is performed using vapor HF in the tool VPE100 from Idonus sàrl, Rouges-Terres 61 2068 Hauterive, Neuchâtel, Switzerland.

In Figure A2a,b a scanning electron microscope (SEM) image of the two devices in this work are reported. The light gray areas are silicon, while the white ones are gold. The darker zones are instead the ones where the material was etched.

## Appendix C. Experimental Set-Up

The electrostatic actuation and capacitive readout are performed by following the scheme reported in Figure A3. The actuation signal is a purely AC signal ($V_{ac}\left(t\right)$) applied to the fixed electrodes named E1 in Figure 2 and Figure 6a. The force (*F*) acting on the device is then proportional to the square of the AC voltage; therefore, the actuation frequency is twice the frequency of the AC signal:

(A2) $$F\left(t\right)=\frac{\partial C_{0}}{\partial x}{\left(V_{ac}cos\left(\omega t\right)\right)}^{2}=\frac{\partial C_{0}}{\partial x}V_{ac}^{2}\frac{\left(1+cos(2\omega t)\right)}{2}.$$

The capacitive readout is performed using a lock-in amplifier (LIA—Zurich instrument model HF2LI from Zurich Instruments AG, Technoparkstrasse 1 8005 Zurich, Switzerland) together with the dedicated transimpedance amplifier. The moving mass (M) is indeed connected to the virtual ground of the transimpedance amplifier (TIA), while the second fixed electrode (E2) is polarized using a DC voltage ($V_{DC}$). The output current follows a sinusoidal function at the MEMS resonance frequency, with a maximum amplitude equal to

(A3) $$I_{MEMS}=\frac{\partial C_{0}}{\partial t}V_{DC}.$$

The current is read by the TIA and converted into a voltage before reaching the input of the LIA. As shown in Figure A3 and mentioned before, the frequency of the current produced by the MEMS is twice the frequency of the excitation voltage (ω). For this reason, the LIA demodulates the signal using the second harmonic of the output signal, thus extracting the amplitude and phase of the MEMS displacement. This technique is effective in eliminating feed-through signals as the electric signal and the MEMS signal are at two different frequencies. Despite such advantage, the signal of the device is still partially covered by parasitic capacitance which couples the second harmonic of the reference signal; therefore, the signal is post-processed in Matlab to eliminate the residual parasitic background. Numerical fitting of the post-processed experimental signal is finally performed to identify the natural frequency and the quality factor *Q* of the devices under test.
